# A Reliable Surgical Procedure for Sinus Floor Augmentation with Antral Pseudocysts

**DOI:** 10.3390/dj9100122

**Published:** 2021-10-18

**Authors:** Yasuhiro Nosaka, Hitomi Nosaka, Yasushi Nakajima, Tadasuke Tanioka, Daniele Botticelli, Shunsuke Baba

**Affiliations:** 1 Department of Oral Implantology, Osaka Dental University, Osaka 540-0008, Japan; y.nakajima@me.com (Y.N.); tanioka.dent@gmail.com (T.T.); baba-s@cc.osaka-dent.ac.jp (S.B.); 2 Nosaka Oral Surgery Clinic, Japan. 2F Chambre-Ashiya, 11-17, Nishiyama-cho, Ashiya 659-0083, Japan; nskshimpuku@gmail.com; 3 ARDEC Academy, 47923 Rimini, Italy; daniele.botticelli@gmail.com

**Keywords:** pseudocyst, maxillary sinus, cone-beam computed tomography, sinus floor augmentation, sinus membrane, maxillary sinus ostium, endoscopy, histology

## Abstract

An antral pseudocyst (AP) is a common well-defined ‘dome-shaped’ faintly radiopaque lesion of the maxillary sinus, and usually does not require treatment in asymptomatic patients. However, when sinus floor augmentation is required to increase bone volume for implant installation, the elevation of the sinus mucosa might drive the AP against the ostium. This might cause its obstruction and, as possible consequence, sinusitis. The purpose of this study was to investigate the clinical and tomographic conditions of APs to identify a predictable cyst removal that might allow a safety sinus floor augmentation. A total of 52 maxillary sinuses in 46 patients (mean age 55.1 years) presenting AP were examined by cone beam computed tomographies (CBCTs). A two-stage approach was applied. At the first surgery, the cystic lesions were further inspected by an endoscope through the antrostomy, and histopathological diagnosis of the removed tissues was carried out. After the confirmation of decrease of the swelling of sinus membrane by CBCT, the sinus floor augmentation was performed, at least four months after cyst removal. The color and transparency of the 86 cystic lesions were classified into 4 types. The whitish transparent cysts were 34 (39.5%), the yellowish transparent cysts were 18 (20.9%), the dark purple transparent cysts were 8 (9.3%) and the milky-white opaque cysts were 26 (30.2%). The contents of the 60 (69.8%) transparent cysts were serous fluid, but those of milky-white cysts were composed of viscous or elastic soft tissues, and the aspiration of the contents was unsuccessful. The analysis of the preoperative CBCT did not provide certainty on the contents of the cystic lesions. All cystic lesions were diagnosed as AP, and an infection was identified in one AP, presenting marked infiltration of the inflammatory cells. Considering the difficulties of performing a correct diagnosis of the AP content by a CBCT analysis, the risk of failure of the surgery that creates severe afflictions to the patients, and the necessity of a histological evaluation of the cyst, a two-stage surgery appeared to be the most reliable procedure.

## 1. Introduction

Maxillary sinus augmentation is a very well documented procedure to restore adequate bone volume in the posterior maxilla, allowing the placement of dental implants [1,2]. However, once an infection occurs, the patients would suffer from sinusitis, accompanied by headache, nasal obstruction, and post-nasal drip. Such complications might compromise bone formation so that a second surgery might be required or, alternatively, the abandonment of the implant treatment. Therefore, a careful examination by cone beam computed tomography (CBCT) is necessary to exclude any lesions in the maxillary sinus.

An antral pseudocyst (AP) is a common disease in the maxillary sinus [3,4]. The AP is a well-defined ‘dome-shaped’ faintly radiopaque lesion at the maxillary sinus cavity and it is detected usually fortuitously by a routine radiographic assessment. As APs are generally asymptomatic in the majority of patients, the treatment is usually unnecessary.

In contrast, when sinus floor augmentation is performed in the presence of an AP, the procedure might significantly reduce the sinus lumen, and cause ostium obstruction after the surgery [5,6,7]. Moreover, when the cyst is perforated, the content of the AP would flow out at surgical field and the contamination might impede bone formation of the augmented area.

The purpose of this study was to investigate the clinical and tomographic conditions of APs to identify a predictable cyst removal that might allow a safety sinus floor augmentation.

## 2. Materials and Methods

### 2.1. Patient Selection and Ethical Statements

The protocol was submitted to and approved by the Japanese society of oral implantology, clinical research ethics committee (MHLW IRB No.11000694), approval No. 2021-3.

From July 2008 to September 2020, a total of 46 patients (30 males and 16 females) ranging in age from 30 to 70 years (mean, 55.1 years) were enrolled in this study and APs were presented in 52 sinuses. All patients had been referred to our clinic by their original dentists for sinus floor augmentation or for an accurate examination of a well-defined ‘dome-shaped’ faintly radiopaque lesion at the maxillary sinus cavity. 

The patients were partially or fully edentulous in the posterior maxilla and required sinus floor augmentation to increase bone volume for implant placement.

The medical histories revealed ten patients with hypertension, four with diabetes, three with heart diseases, and three with allergic rhinitis. All of these diseases were well controlled by pharmacological treatments. Thirteen patients (28.3%) were smokers.

### 2.2. CBCT Evaluation

**Figure 1:**
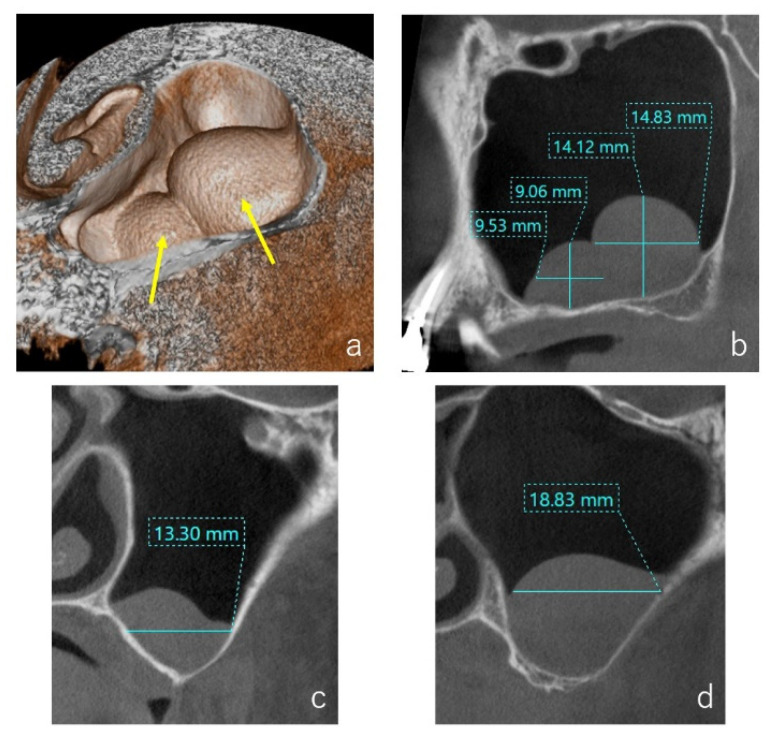
Case: 62 years old female. Two cystic lesions were observed in the left maxillary sinus by the volume rendering image ((**a**), yellow arrows). A sagittal section of the CT image showed two well-defined ‘dome-shaped’ faintly radiopaque lesions, and the size of mesiodistal and vertical lengths were measured (**b**). On the buccolingual section of the CT image, the buccolingual length of the medial (**c**) and distal (**d**) cystic lesions were measured.

The CBCT (3D Accuitomo, Morita, Kyoto, Japan, 80 kV, 5.0 mA) evaluations were performed before the surgery and immediately, one week and three months after the surgery. The numbers of cystic lesions were counted (Figure 1a, yellow arrows) and the size was measured three-dimensionally (mesiodistal × vertical × buccolingual length) by the CBCT (Figure 1b–d).

### 2.3. Surgical Procedure

**Figure 2:**
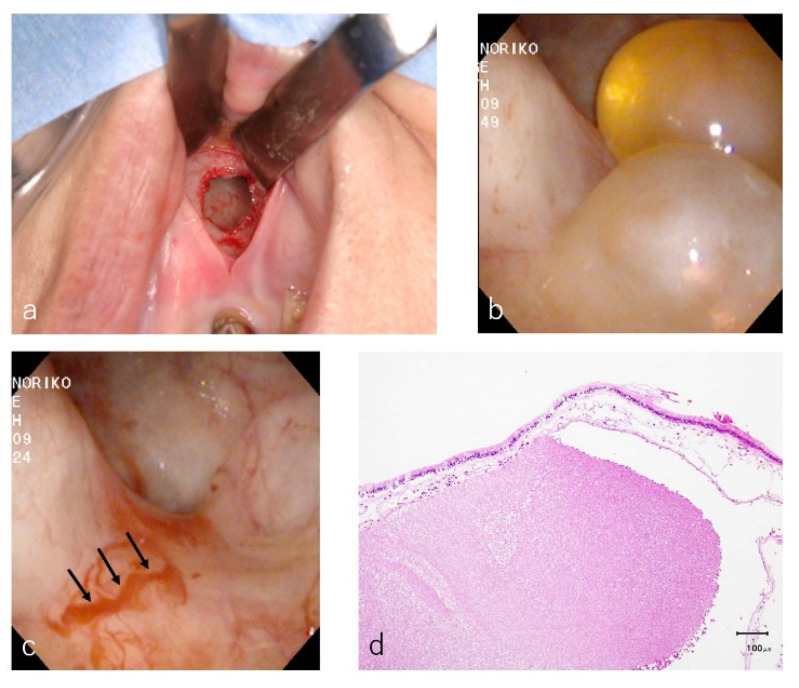
The same case as Figure 1. After a vertical incision, an intentional perforation of the sinus membrane was performed through the window (**a**). Two yellowish transparent cysts were observed by the endoscope (**b**) and were removed at the superior layer of the periosteum which contained blood vessel ((**c**), black arrows). Histological examination of a hematoxylin and eosin staining slide demonstrated a single layer of ciliated columnar epithelium and connective tissue with slight degree of inflammatory cells. Among the connective tissue, eosinophilic content was observed, and the diagnosis was an antral pseudocyst (**d**).

The surgeries were performed by the same oral surgeon under local anesthesia with intravenous sedation. A vertical incision was made at the vestibular sulcus and a mucoperiosteal flap was elevated to expose the surgical site. An antrostomy with a diameter of approximately 10 mm was made at the frontal wall by using diamond round bur, and the intentional perforation of the sinus membrane was performed (Figure 2a). 

**Figure 3:**
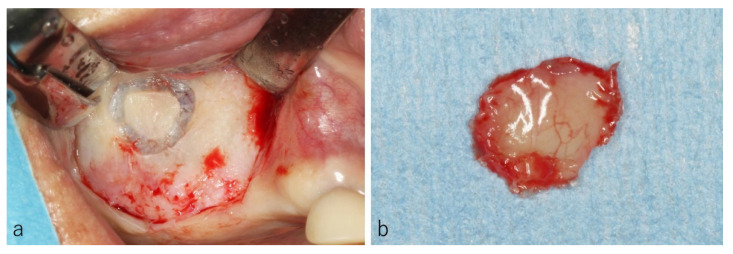
Case: 61 years old male. The osteotomy was performed at the frontal wall (**a**) and the bone segment was removed along with maxillary sinus membrane (**b**).

In five sites, the osteotomies were performed at the frontal wall and the bone segments were removed along with maxillary sinus membrane to evaluate histological structures of the sinus membrane (Figure 3a,b).

**Figure 4:**
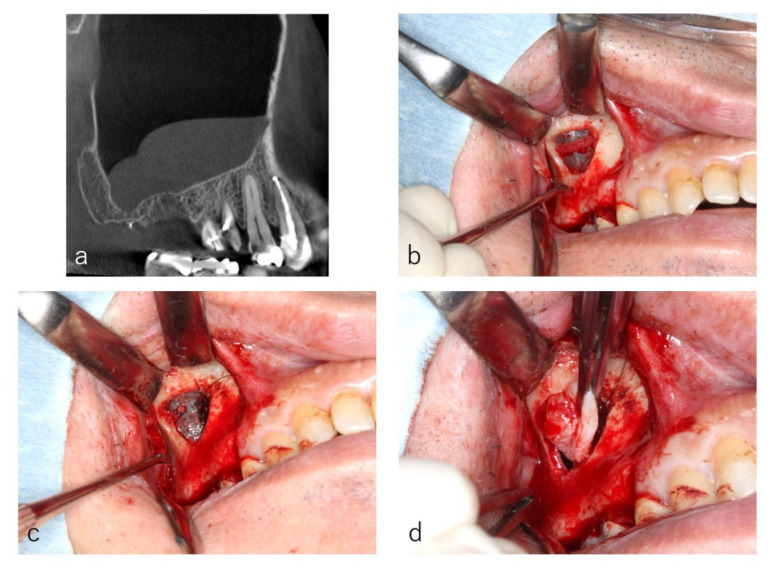
Case: 55 years old male. A sagittal section of the CT image showed two well-defined ‘dome-shaped’ faintly radiopaque lesions (**a**). At the window, the posterior superior alveolar artery was denuded (**b**), and the blood vessel was ligated and amputated (**c**). The cystic lesions were enucleated through the window (**d**).

When a posterior superior alveolar artery ran across the frontal wall, a mid-crestal incision was added to secure a sufficient field of view. The blood vessel was denuded (Figure 4b), ligated and amputated to prevent bleeding during surgery (Figure 4c).

**Figure 5:**
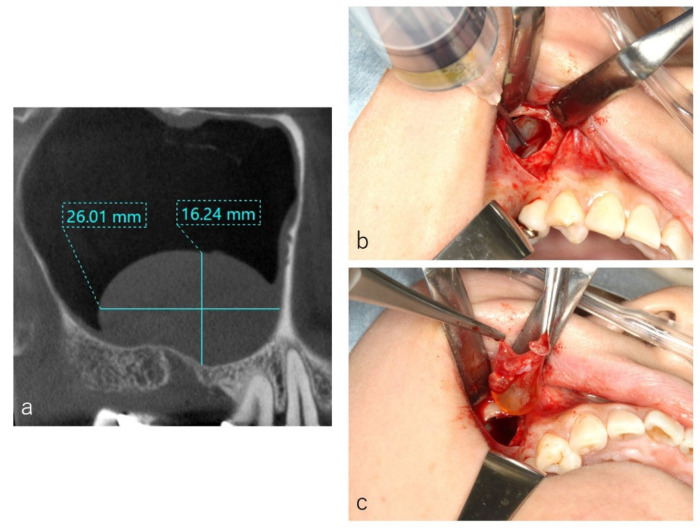
Case: 44 years old female. As the medio-distal size was 26.0 mm (**a**), the preliminary suction of the contents of cystic lesion was performed with a fine 18-gauge needle (**b**). The wall of the cystic lesion was clamped with forceps and herniated through the window with gentle outward traction (**c**).

The cystic lesions were inspected by the naked eye and by endoscope through the window (Figure 2b). When the sizes of cystic lesions were large (more than 20 mm), a preliminary aspiration of the content of cystic lesions was performed with a fine 18-gauge needle to reduce the volume of the cysts to facilitate the clamping of the tissues (Figure 5a,b). The wall of the cystic lesion was clamped with forceps and herniated through the window with a gentle outward traction trying to preserve the integrity of the periosteum underneath the lesion (Figure 5c). 

After confirmation by the endoscope of the hemostasis (Figure 2c), the antrostomy was covered with a resorbable collagen membrane to reduce blood inflow to the sinus cavity, and the mucoperiosteal flap was closed. As an antibiotic, Azithromycin hydrate (500 mg) was taken 1 h before the surgery and once a day following two days. Loxoprofen sodium hydrate (60 mg) were prescribed for single use as a pain killer.

All patients were warned about the possibility of postoperative intermittent epistaxis for a few days and were instructed not to blow their nose for the first 14 days to avoid the wound dehiscence due to the momentary high pressure of the sinus cavity. All of the removed tissues were sent to the routine histopathologic examination for diagnosis (Figure 2d).

## 3. Results

### 3.1. Histology of the Frontal Wall

**Figure 6:**
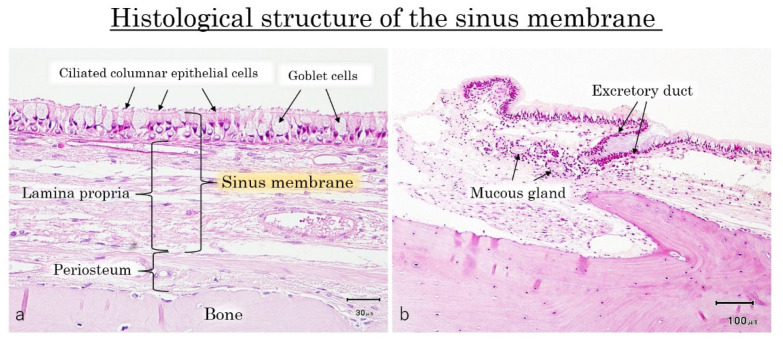
The sinus membrane was consisted of two layers. One was the surface layer with ciliated columnar epithelium cells and goblet cells, and the another was the lamina propria with loose connective tissue. Beneath the sinus membrane, the periosteum containing blood vessels was existed at the surface of bone (**a**). In this specimen, the mucous glands were observed in the lamina propria, and the excretory duct containing mucous was extended to the surface of sinus membrane (**b**).

The sinus mucosa showed a normal structure composed by two layers, the ciliated columnar epithelium with goblet cells, and the lamina propria with loose connective tissue (Figure 6a). Beneath the sinus membrane, the periosteum containing blood vessels connected the membrane to the bone surface.

In the present study, only in one site out of five windows examined mucous glands were observed in the lamina propria, and the excretory duct containing mucous was extended to the surface of sinus membrane (Figure 6b).

### 3.2. CBCT Evaluation of the Cystic Lesions

**Figure 7:**
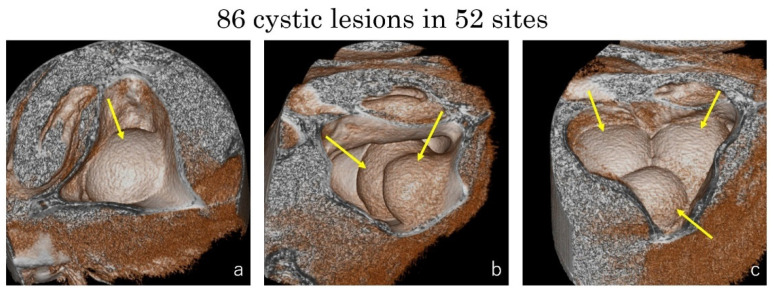
The numbers of cystic lesion in 52 sites were counted by the volume rendering images (yellow arrows). (**a**) One cyst in twenty-six sinuses (50%); (**b**) two cysts in twenty sinuses (38.5%); (**c**) three or more cysts in six sinuses (11.5%).

Eighty-six cystic lesions were observed in the fifty-two sinuses assessed in forty-six patients. Six patients (13.0%) presented cysts in both sinuses. In 26 sinuses (50.0%) a single cyst was observed, in 20 sinuses (38.5%) 2 cysts were assessed, and 6 sinuses (11.5%) presented 3 or more cysts (Figure 7). The smallest size of the cystic lesion was 3.3 × 5.1 × 2.8 mm, the biggest one was 30.6 × 25.1 × 24.1 mm. The mean size was 14.5 (±6.3) × 12.3 (±5.1) × 13.0 (±4.5) mm.

**Figure 8:**
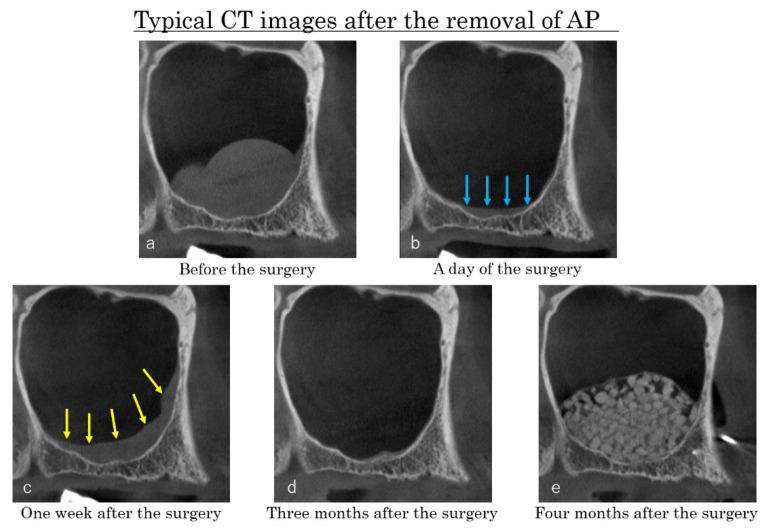
Case: 62 years old female. Two APs were detected on the CBCT before surgery (**a**). The cysts were removed, and the flat shaped faint opacity at the bottom of maxillary sinus was observed on the CBCT taken immediately after the surgery ((**b**), blue arrows). One week after the surgery, a post-operative swelling of the sinus membrane was observed ((**c**), yellow arrows). The swelling had disappeared three months after the surgery (**d**), and the sinus floor augmentation was performed successfully four months after the removal of Aps (**e**).

On the CBCT images taken immediately after the surgery, a flat shaped faint opacity at the bottom of maxillary sinus was observed in 47 sites (90.4%), composed of a pool of blood and physiologic saline (Figure 8b, blue arrows). In the CBCTs taken 1 week after surgery, a post-operative swelling of the sinus membrane (Figure 8c, yellow arrows) was observed in 50 sites (96.2%).

Three months after the surgery, the post-operative swelling of the sinus membrane had decreased under 2 mm in forty-six sites (88.5%), and the sinus floor augmentations were performed at least four months after cyst removal (Figure 8d,e). As the bone material for sinus floor augmentation, the beta-tricalcium phosphate (β-TCP) granules (OSferion, Olympus Terumo Biomaterials, Tokyo, Japan) were used in all patients.

**Figure 9:**
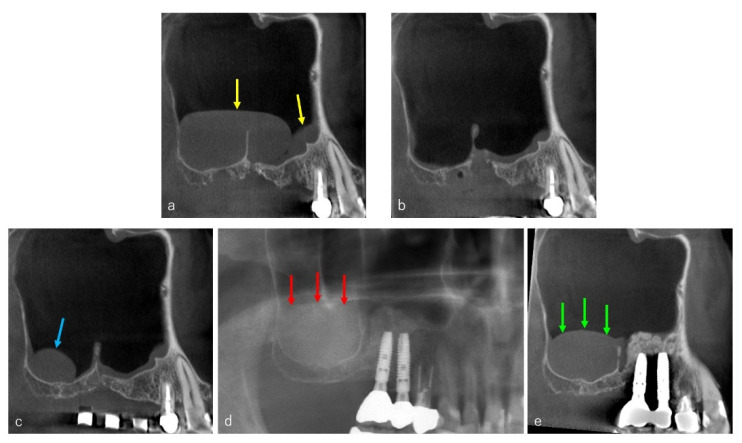
Case: 60 years old male. Two APs were observed, and the posterior AP existed across the septum (**a**), yellow arrows). They were removed, and the curettages of the extraction sockets were performed simultaneously (**b**). Three months after the removal, a relapse of AP had been occurred at the posterior area of septum ((**c**), blue arrow). Eight months after the removal, the sinus floor augmentation was performed only at the medial area of septum, and two implants were placed at the augmented area. Two point five years after the removal of APs, a panoramic radiograph ((**d**), red arrows) and a sagittal section CT image ((**e**), green arrow) showed the increased AP at the posterior area of septum.

A relapse of AP occurred in one site (1.9%). However, the implant treatment was completed after the sinus floor augmentation (Figure 9).

### 3.3. Types of Colors and Contents of the Cystic Lesions

**Figure 10:**
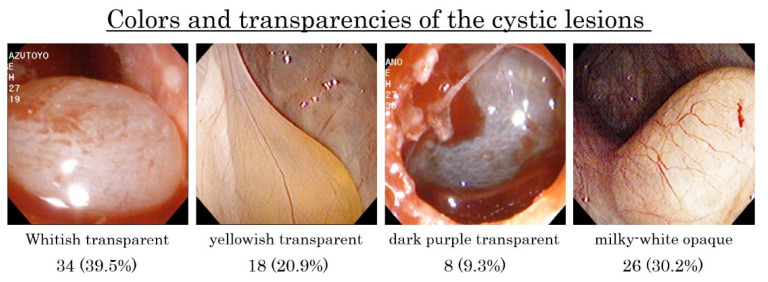
The colors and transparencies of the cystic lesions were classified into four types by the visual and endoscopic examinations. The 60 cystic lesions (69.8%) were transparent and 26 (30.2%) were opaque.

The colors and transparencies of cystic lesions were classified into four types (Figure 10). 

**Figure 11:**
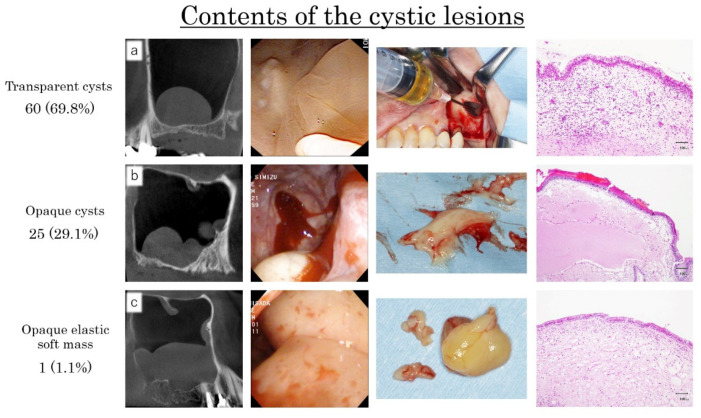
(**a**): 68 years old female. The content of a yellowish transparent cystic lesion was serous fluid and could be aspirated smoothly with a fine 18-gauge needle. (**b**): 48 years old male. The content of a white opaque cystic lesion was milky-white viscous, and it was difficult to be aspirated. (**c**): 30 years old female. The content of a white opaque cystic lesion was organized and exhibited elastic soft mass as with a tumor. All of the histological specimens were diagnosed as APs with moderate infiltration of inflammatory cells.

The whitish transparent cysts were 34 (39.5%), the yellowish transparent cysts were 18 (20.9%), the dark purple transparent cysts were 8 (9.3%), and the milky-white opaque cysts were 26 (30.2%). In the transparent cystic lesions, the contents were serous fluid and could be aspirated smoothly with a fine 18-gauge needle (Figure 11a).

However, in 25 (29.1%) opaque cystic lesions, the contents were viscous and difficult to be aspirated (Figure 11b) while, in one, the content was organized and exhibited elastic soft mass as a tumor (Figure 11c).

In all sites, it was difficult to assess the contents of the cystic lesions by preoperative CBCT. All the histological specimens were diagnosed as APs with moderate infiltration of inflammatory cells.

### 3.4. Histopathological Evaluations of the Cystic Lesions

The microscopic examinations of cystic lesions revealed the laying of ciliated columnar epithelial cells and connective tissues with some inflammatory cells (lymphocytes and plasma cells), and were diagnosed as AP (Figure 2 and Figure 11). 

**Figure 12:**
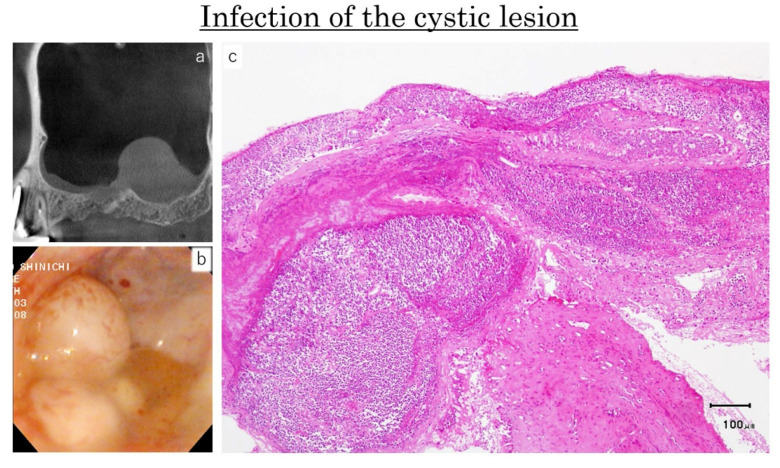
Case: 49 years old male. At the CBCT evaluation, the presence of a single cyst was assessed (**a**). However, at the endoscopic evaluation, one transparent and three opaque small cysts were detected (**b**). The histological specimen of the opaque cyst showed marked infiltration of the inflammatory cells and the transformation of the ciliated columnar epithelial cells into the squamous cells (**c**).

In one site, one transparent and three opaque small cysts were observed (Figure 12a,b). The histological specimen showed marked infiltration of the inflammatory cells and the transformation of ciliated columnar epithelial cells into the squamous cells (Figure 12c).

### 3.5. Surgery and Clinical Progress

In eight sites (15.4%), the posterior superior alveolar artery ran across the frontal wall where the window was scheduled to be made. A mid-crestal incisions were added, and the blood vessels were ligated and amputated. No postoperative bleeding which needed hemostatic treatment occurred.

In the few days of post-operative period, the patients complained slight pain and facial swelling, and small nose bleeding was observed in 49 cases (94.2%). All stiches were removed eight days after the surgery and local infection did not occur in any patient.

In 28 sinuses (53.8%) out of 52, sinus floor augmentations were performed 4 to 6 months after the removal of APs in our clinic. In 16 sinuses (30.8%), sinus surgeries had been carried out more than 7 months after the removal for convenience of reservation. In 8 sinuses (15.4%), the sinus floor augmentations were performed at their original clinics.

**Figure 13:**
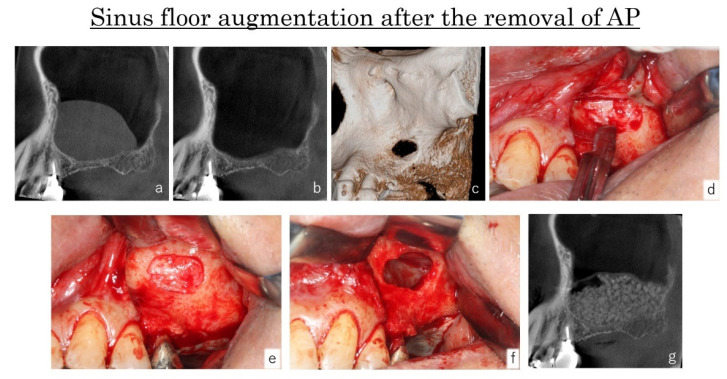
Case: 48 years old male. Three months after the removal of AP, the swelling of the sinus membrane had disappeared (**a**,**b**). As the bone defect at the antrostomy was still present, the dissection of the scar tissues between the sinus membrane and oral mucosa was performed during the elevation of the muco-periosteal flap (**c**–**e**). The sinus floor augmentation was completed without perforations of the sinus membrane (**f**) and bone materials were fill at the augmented area (**g**).

At the time of sinus floor augmentation, the bone defect of the antrostomy performed at the previous surgical session was still present in all sites (Figure 13c). Therefore, during the elevation of muco-periosteal flap, the dissection of the scar tissues between the sinus membrane and the oral mucosa was necessary to avoid sinus mucosa perforation (Figure 13 d,e).

In 8 sinuses (18.2%) of 44, the ruptures of sinus membrane had occurred due to the complex septa. The collagen membranes (Terudermis, Olympus Terumo Biomaterials, Tokyo, Japan) were putted on the surface of the periosteum layer underneath sinus membrane. 

The processes of the sinus floor augmentations were good, and no infection had occurred after the surgery (Figure 13d).

## 4. Discussion

The histological specimens taken from the lateral wall of the sinus showed the classical two layers of the sinus membrane, namely the ciliated pseudostratified columnar epithelium containing goblet cells, and the lamina propria that was in close contact with the periosteum. This confirmed that sinus membrane and periosteum are both included in the elevation procedure. Furthermore, the swelling of sinus membrane was due to the edema within the lamina propria, with inflammatory cells infiltration and hyperplasia of the capillary blood vessels. However, the thickness of epithelium cells, goblet cells, and periosteum were almost unchanged.

The sinus membrane swells very easily due to the odontogenic focuses at the bottom of maxillary sinus, and the inflammation of the sinus membrane sometimes demonstrate dome-shaped faintly radiopaque swelling. These swelling of the sinus membrane usually disappear three months after the treatment of the focuses. Therefore, the treatment of the odontogenic focuses should be performed before CT evaluation to avoid misdiagnosis. Furthermore, when the bone resorption was observed, the biopsy would be required before removal of cystic lesion to exclude the possibility of tumor.

Even though the AP is a common finding in the maxillary sinus, the treatment approach for sinus floor augmentation in the presence of AP is still a controversial topic given that the AP is rarely removed in asymptomatic patients. According to our data, 13 patients (28.3%) were smokers, and it was suggested that there is no connection between smoking and APs.

In the histological specimen of the lateral wall, the mucous glands and excretory duct were observed in the lamina propria. It was suggested that AP might occur due to the obstruction of the excretory duct, and consequently the mucous material secreted by the mucous gland might deposited inside the duct [8]. Therefore, AP would be situated at the lamina propria layer, and the content is surrounded by ciliated columnar epithelial cells, connective tissue of lamina propria and periosteum. So, the removal of AP means marsupialization, that is, the ciliated columnar epithelium and connective tissue should be removed without injury the periosteum to avoid bleeding.

Various treatment methods of sinus floor augmentation with APs had been advocated, and they were roughly categorized into four types [9,10,11,12,13].

1. Sinus floor augmentation without the removal of the AP 

This method exposes to the risk of maxillary sinusitis due to the obstruction of ostium by the coronal reposition of AP after the elevation of sinus membrane. If the periosteum was teared during the surgery, the contamination of the surgical area would occur due to the flow out of the content of AP.

Depending upon the location and numbers, AP under 10 mm of size be the candidate for this method [9,10]. However, the final diagnoses of the cystic lesions cannot be obtained.

2. Sinus floor augmentation with only the aspiration of AP

This method seemed to be an easy procedure to reduce the volume of AP [11]. However, according to the data from the present study, 26 (30.2%) out of the 86 APs evaluated demonstrated milky-white opaque lesions and the contents of APs were viscous or elastic soft tissue. Therefore, the aspirations of the contents of APs were unsuccessful in approximately 30% of APs. This, in turn, means that the aspiration method alone could not reach the goal of eliminating the cyst contents. More important was that it was impossible to specify the character of the contents of APs by the preoperative CBCT images. Moreover, the aspiration of contents is not a curative treatment and leave the focuses such as obstructed excretory duct and related mucous glands. A relapse of AP might induce sinusitis due to the obstruction of the ostium for the higher position of the cyst compared to the original location. Still, in this method the final diagnoses of the cystic lesions were unavailable.

3. Simultaneous sinus floor augmentation with removal of AP

This method makes it possible to shorten the period of implant treatment and reduces the surgical sessions [12]. Moreover, with this approach, the relapse of AP would be unusual, and the final diagnosis might be obtained by the histopathological examinations.

However, when more than two APs are present, the surgical procedure might become difficult. According to the data reported in the present study, single APs were observed in 26 sites (50.0%) among 52 sites, and multiple APs were detected in other 26 sites (50.0%). Furthermore, the presence of posterior alveolar artery complicated the surgery so that in 8 sinuses (15.4%) out of 52, the ligations of the posterior alveolar artery were carried out. When adopting this surgical approach, size, numbers, position of APs and estimation of the position of the posterior alveolar artery with respect to that of the antrostomy should be carefully investigated before surgery. 

The APs usually present a dome-shaped sessile lesion, and the base of lesion is difficult to be detected by the pre-operative CBCT images. Therefore, the size of antrostomy sometimes becomes large to remove the base of APs and it make impossible to create the window for sinus floor augmentation. After the removal of AP, the periosteum underneath the AP has lost the backing layer of sinus membrane and becomes weaker. So, the small perforation of periosteum develops larger easily and the risk of the interruption of surgery would be increased.

Therefore, this method should be performed in case of single AP with small base. A high-level skill of the surgeon is required.

4. Two-stage surgery of AP removal and sinus floor augmentation

As a two-stage surgery impose physical burdens on the patients, the minimum invasive surgery is required for the removal of AP [13]. Nevertheless, position, size, and number of APs influence the flap design and a mid-crestal incision might be required. The use of an endoscope was especially useful to observe the APs through the small window and to confirm the full removal of APs and a good hemostasis. 

The infection of an AP was recognized in one case, and this was not detected prior the surgery by the pre-operative CBCT images or clinical aspects. In this case, a two-stage surgery was a reliable and safety procedure on the sinus floor augmentation.

One week after the removal of AP, a post-operative swelling of sinus membrane was observed at the CBCT. This post operative swelling of the sinus membrane would occur due to the traumatic stimulation of the surgery and it was documented in several references [14,15,16,17,18]. Three months after the surgery, the swelling of the sinus membrane had decreased spontaneously [14]. It has also been documented that the post-surgical edema might reach the ostium and infundibula, causing their obstruction also in absence of pathologies within the sinus [19,20]. To avoid juxta- and post-surgical complications, an accurate evaluation of various anatomical and clinical parameters should be performed on CBCTs before undertake sinus floor augmentation [21,22].

The regression of the post-surgical edema observed also in the present study suggests that the sinus membrane at the surface of AP had been regenerated and the function of the ciliated columnar epithelium cells for clearance of the maxillary sinus had resumed. The periosteum underneath AP might assure again a strong contact with the regenerated sinus membrane. This outcome suggests that the sinus floor augmentation should be performed after the confirmation of the decreased swelling of the sinus membrane.

When performing the sinus floor augmentation, the position of the first antrostomy should be estimated. During the elevation of the muco-periosteal flap, an accurate dissection of the scar tissues between the sinus mucosa and oral mucosa is necessary to avoid perforations.

## 5. Conclusions

Considering the difficulties of performing a correct diagnosis of the AP content by a CBCT analysis, the risk of failure of the surgery that creates severe afflictions to the patients, and the necessity of a histological evaluation of the cyst, a two-stage surgery appeared to be the most reliable procedure. This approach includes AP removal and, in a subsequent surgical session, the sinus floor augmentation.

## Data Availability

The data are available on reasonable request.
